# Efficacy of carrimycin against *Mycobacterium avium* complex *in vitro* and *in vivo*

**DOI:** 10.1128/spectrum.02422-24

**Published:** 2025-08-08

**Authors:** Zimo Wang, Xiaodong Li, Weiyan Zhang, Lei Fu, Xinda Li, Xiaoyou Chen, Yu Lu

**Affiliations:** 1 Beijing Key Laboratory of Drug Resistance Tuberculosis Research, Beijing Chest Hospital, Capital Medical University, Beijing Tuberculosis and Thoracic Tumor Research Institute 117550, Beijing, China; 2 Beijing Ditan Hospital, Capital Medical University 12517, Beijing, China; City of Hope Department of Pathology, Duarte, California, USA

**Keywords:** non-tuberculous mycobacteria, *Mycobacterium avium *complex, carrimycin, BALB/c mice

## Abstract

*Mycobacterium avium* complex (MAC) is one of the principal pathogenic strains among nontuberculous *mycobacteria*. Current antibiotics have poor microbiological responses, and repurposed antibiotics are a way to meet clinical needs. Herein, the anti-MAC activity of carrimycin (CAM), a new macrolide, was evaluated *in* vitro and *in vivo*. Antimicrobial susceptibility testing was performed on two reference strains and 15 clinical isolates of MAC. The synergy between CAM and nine clinically used antibiotics was determined using a checkerboard assay. The activity of CAM against MAC residing inside macrophages and BALB/c mice were also evaluated. Although CAM exhibited weak anti-MAC activity *in vitro*, with MICs over 8 mg/L, the *in vivo* activity was potent. CAM treatment significantly reduced bacillary load, alleviated the severity of injury, and improved total T lymphocyte numbers in organs. Additionally, a synergy between CAM and clofazimine was found *in* vitro and in *vivo*. No antagonism was found between CAM and other eight antibiotics used in this study. In conclusion, CAM is a potential candidate and should be studied further in clinical trials.

**IMPORTANCE:**

The treatment of *Mycobacterium avium* complex (MAC) infections is a formidable challenge worldwide due to lack of effective drugs. In this paper, we firstly reported a promising candidate drug carrimycin (CAM). For *M. intracellulare*, the activity of CAM was compared with classical drug azithromycin (AZM). For *M. avium*, CAM also showed potent activity *in vivo*. Additionally, the combination of CAM and CFZ reduced more bacillary load than single drugs *in vivo*. Our study also indicated that we should take pharmacokinetic characteristics and drug interactions into account when evaluating the efficacy of drugs.

## INTRODUCTION

Non-tuberculous *mycobacteria* (NTM) encompasses a large number of *Mycobacterium* species that are mainly environmental opportunistic pathogens. However, there is growing awareness of the emergence of NTM as causative agents of infections worldwide. One of the principal pathogenic strains is *Mycobacterium avium* complex (MAC), especially *M. avium* and *M. intracellulare*. MAC infections unusually occur in immunocompromised individuals and those with underlying lung diseases, such as bronchiectasis, cystic fibrosis, chronic obstructive pulmonary disease, and so on (1). It poses a significant global public health challenge, leading to substantial morbidity and mortality.

Owning to poor culture conversion, high rates of recurrence and reinfection, and severe side effects, the treatment of MAC infections is a formidable challenge. According to the American Thoracic Society and the Infectious Diseases Society of America (ATS/IDSA) guideline, current guideline-based therapy (GBT), including clarithromycin/azithromycin, ethambutol, and rifampicin. It is universally acknowledged that macrolides are the cornerstone of therapy, and the treatment outcomes have relationships with the susceptibility of macrolides. Azithromycin enjoys extensive application due to superior tolerability, fewer drug interactions, lower pill burden, and without compromising efficacy when compared with clarithromycin. An azithromycin-based regimen was more likely to continue than a clarithromycin-based regimen (2). However, AZM is a broad-spectrum antibiotic with extensive clinical utilization, and the resistant strains are increasing gradually. Hence, identifying and developing novel macrolides would be an effective strategy for urgent treatment of MAC infections.

Carrimycin (CAM; trade name: Bite; formerly shengjimycin and bitespiramycin) was approved by the Chinese Food and Drug Administration (CFDA) for the treatment of acute tracheal bronchitis and acute sinusitis. It is a novel macrolide antibiotic produced by genetically engineered *Streptomyces spiramyceticus* harboring a 4″-O-isovaleryltransferase gene (ist) from *Streptomyces thermotolerans*. The components of CAM are isovalerylspiramycin (ISP) I–III, and a certain amount of (iso) butyryl/propionyl/acetylspiramycin III and (iso) butyryl/propionyl/acetylspiramycin II. CAM is an excellent antibiotic that can inhibit bacterial protein synthesis by blocking the activity of peptidyl transferase in the 50s ribosomes (3). CAM has exhibited notable efficacy in treating respiratory bacterial infections, with clinical outcomes and safety profiles no inferior to AZM.

CAM has activity against *Mycobacterium tuberculosis*. Using CAM in a patient with acute promyelocytic leukemia (APL) combined with pulmonary *tuberculosis* (PTB), who could not tolerate routine anti-*tuberculosis* drugs, achieved a satisfactory result (4). The effectiveness of CAM in the treatment of *Mycobacterium abscessus* pulmonary diseases is ongoing (ChiCTR2100054540). Notwithstanding these applications, its efficacy in the treatment of MAC infections remains unexamined. Therefore, we explore the anti-MAC activity of CAM and firstly find that CAM has potent activity against MAC *in vivo*. We highly recommend conducting additional clinical trials for CAM.

## RESULTS

### CAM has activity against MAC

A total of 15 MAC clinical isolates (8 *M*. *avium* and 7 *M*. *intracellulare*) and two MAC reference strains (ATCC 12478 and ATCC 13950) were collected. The MIC values in Middlebrook 7H9 broth and CAMHB were similar, with the disparity falling within a twofold range. CAM and AZM exhibited comparable anti-MAC activity. The MICs of CAM and AZM in *M. avium* ranged from 8 to >32 mg/L, while in *M. intracellulare* also ranged from 8 to >32 mg/L (Table 1).

**TABLE 1:** MICs of CAM and AZM against MAC^*a*^

Strain	MIC (mg/L) in 7H9 broth		MIC (mg/L) in CAMHB broth	
	CAM	AZM	CAM	AZM
*M. avium*				
ATCC 25291	16	8	8	8
2-9	16	16	16	16
2-11	16	8	16	16
2-15	16	8	16	16
2-16	16	16	16	8
8-1	16	16	16	16
8-9	32	16	32	32
2-8	>32	>32	32	32
8-15	>32	>32	>32	>32
*M. intracellulare*				
ATCC 13950	32	32	>32	>32
8-17	16	16	32	16
8-13	16	8	16	8
8-14	16	16	16	32
2-4	>32	>32	>32	32
2-5	>32	>32	>32	>32
8-12	>32	>32	>32	>32
8-16	>32	16	>32	16

^*a*^ CAM, carrimycin. AZM, azithromycin.

### CAM inhibits the growth of intracellular MAC

**Fig 1:**
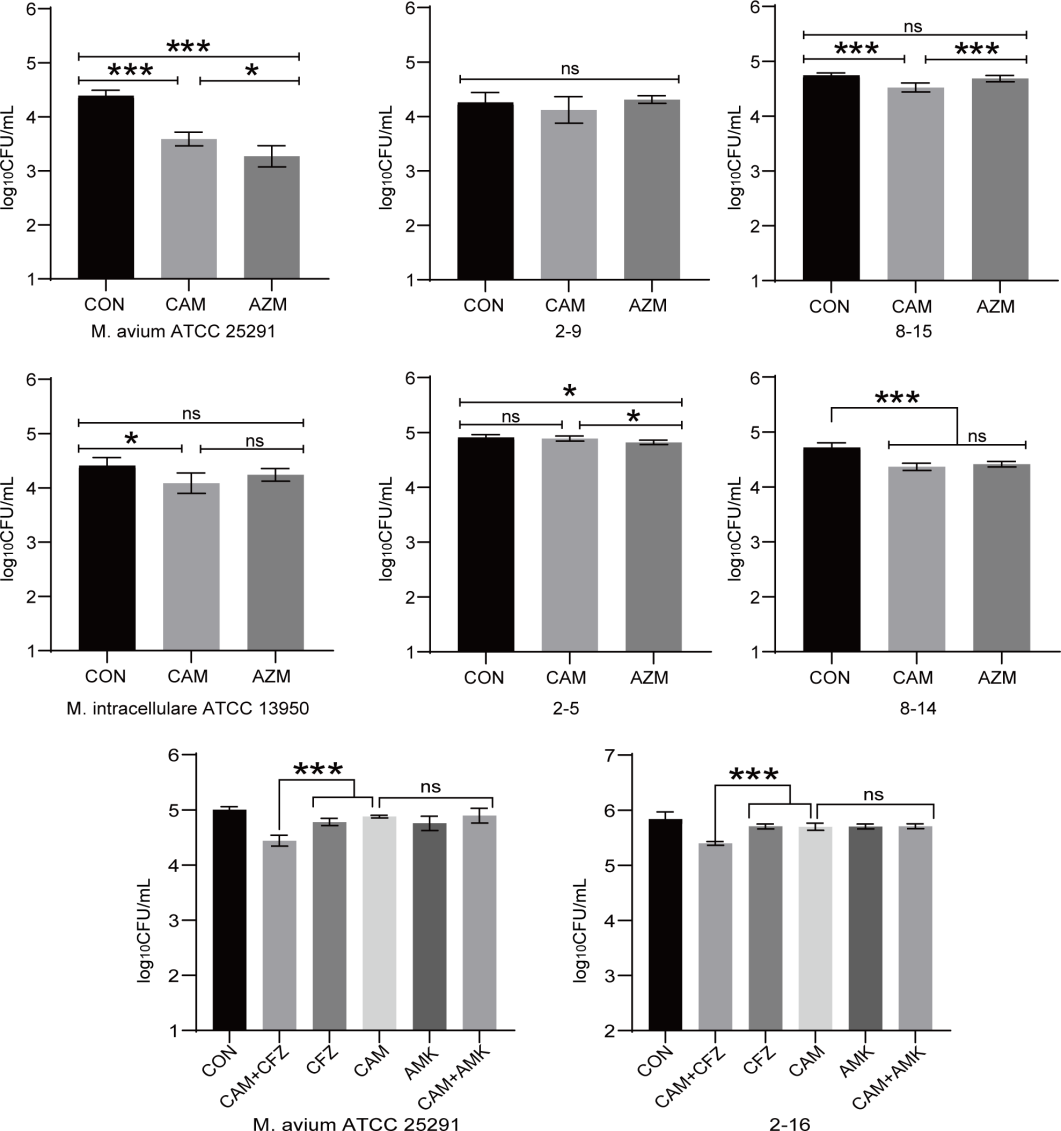
Intracellular activity of CAM and AZM against NTM strains. Data are presented as means ± standard deviations of 6 replicates. *M. avium* ATCC 25291 and *M. intracellulare* ATCC 13950 were MAC reference strains. 2-9, 2-16, and 8-15 were *M. avium* clinical isolates. 2-5 and 8-14 were *M. intracellular* clinical isolates. CON, control. CAM, carrimycin. AZM, azithromycin. CFZ, clofazimine. AMK, amikacin. ns, *P* > 0.05. *, *P* < 0.05.***, *P* < 0.001*.*

The intracellular antimicrobial activity of CAM and AZM was assessed in 2 reference strains and randomly selected 4 clinical isolates at the concentration of 2 mg/L. In *M. avium* reference strain (ATCC 12478), CAM and AZM reduced intracellular bacterial load by 0.80 and 1.11 log_10_ CFU/mL (*P* < 0.001). In *M. intracellulare* reference strain (ATCC 13950), CAM and AZM reduced intracellular bacterial load by 0.32 and 0.16 log_10_ CFU/mL (*P*_CAM_ = 0.012, *P*_AZM_ = 0.218). CAM and AZM also reduced intracellular bacterial load in clinical isolates (Fig. 1).

### CAM is potently active against MAC *in vivo*

**Fig 2:**
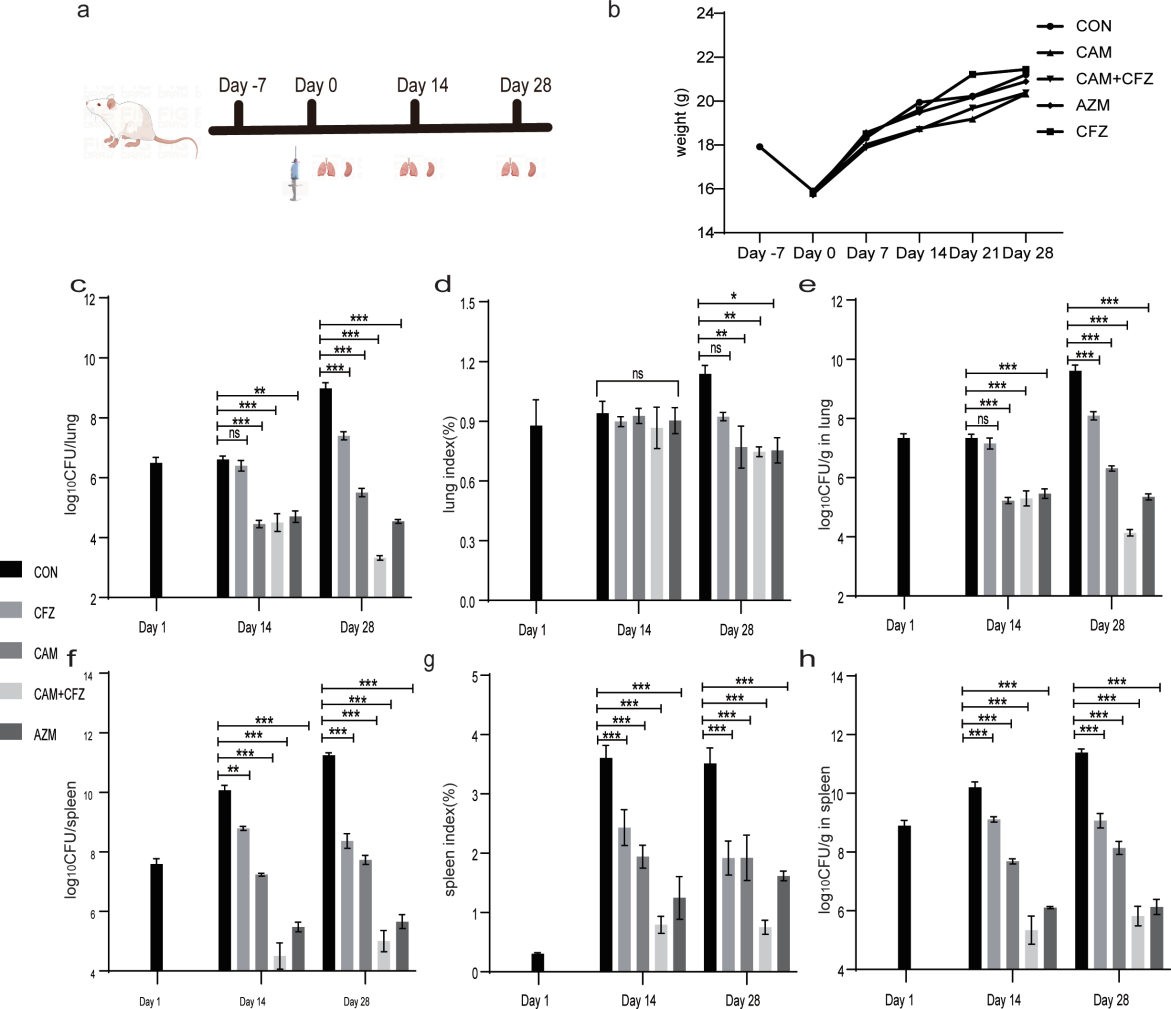
*In vivo* efficacy of CAM and AZM against *M. avium* (ATCC 25291). (**a**) The process of the whole experiment. Briefly, BALB/c mice were continuously administered 5 mg/kg dexamethasone from day −7 to day −1 and then infected by tail vein injection at day 0. Mice were dissected for CFU counting when CAM or AZM treatment reached 2 and 4 weeks. (**b**) Body weight. (**c**) Log_10_ CFU/ in lung. (**d**) Lung index. (**e**) Log_10_ CFU/g in lung. (**f**) Log_10_ CFU/ in spleen. (**g**) Spleen index. (**h**) Log_10_ CFU/g in spleen. The dosage of CAM, AZM, and CFZ was 100, 100, and 25 mg/kg, respectively. Data are represented as mean ± standard deviation. *n* = 6. ns, *P* > 0.05. *, *P* ≤ 0.05. **, *P* ≤ 0.01. ***, *P* ≤ 0.001.

**Fig 3:**
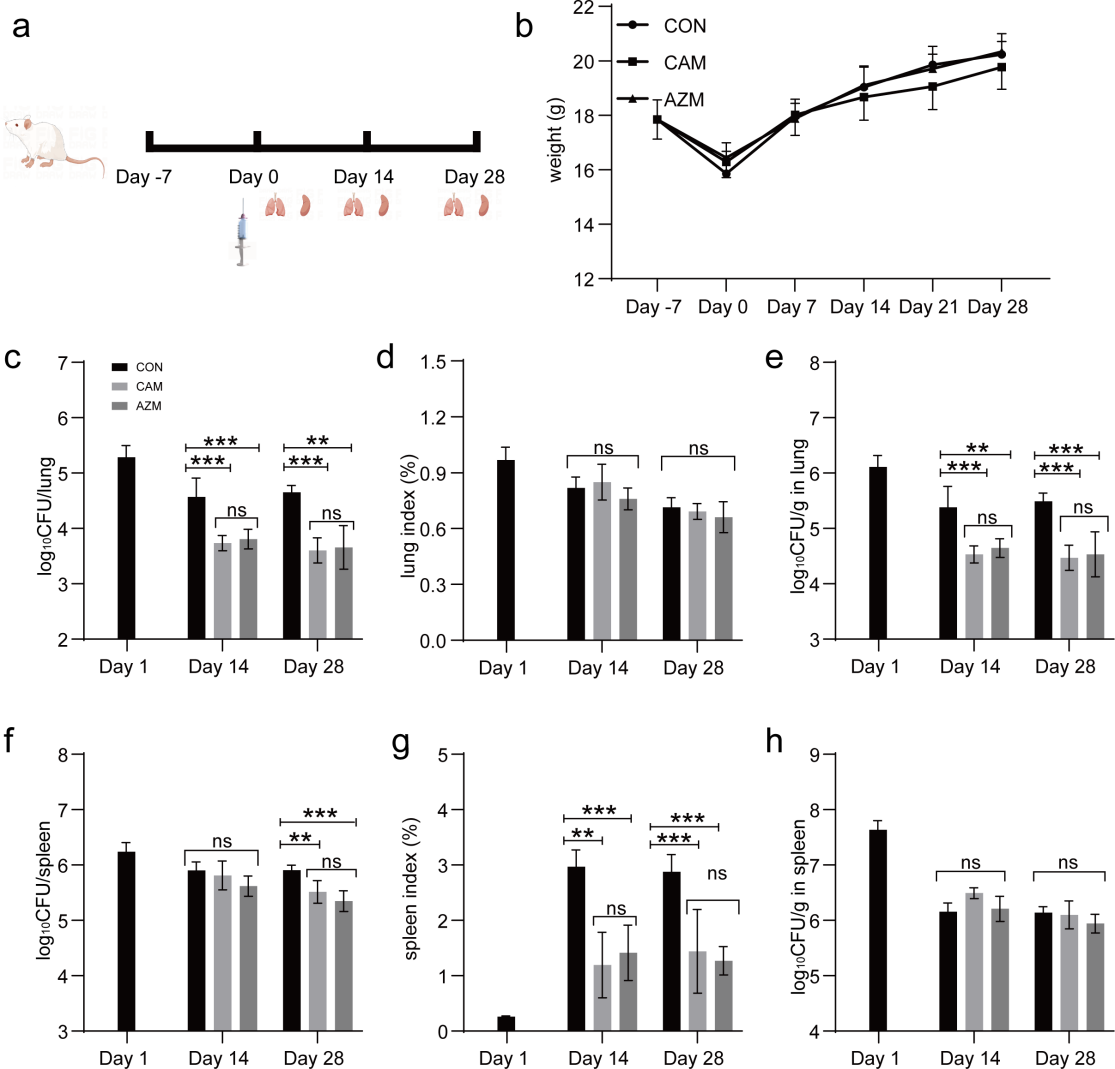
*In vivo* efficacy of CAM against *M. intracellulare* (ATCC 13950). (**a**) The process of the whole experiment. (**b**) Body weight. (**c**) Log_10_ CFU/lung. (**d**) Lung index. (**e**) Log_10_ CFU/g in lung. (**f**) Log_10_ CFU/spleen. (**g**) Spleen index. (**h**) Log_10_ CFU/g in spleen. The dosage of CAM and AZM was 100 and 100 mg/kg. Data are represented as mean ± standard deviation. *n* = 6. ns, *P* > 0.05. *, *P* ≤ 0.05. **, *P* ≤ 0.01. ***, *P* ≤ 0.001.

The efficacy of CAM was robust against *M. avium* (ATCC 25291) and *M. intracellulare* (ATCC13950) in BALB/c mouse models. For *M. avium* (ATCC 25291), the efficacy of CAM was slightly inferior to AZM. CAM and AZM treatment reduced the bacillary load in lungs by 2.15 and 1.91 log_10_ CFU (*P* < 0.001), and in spleens by 2.82 and 4.58 log_10_ CFU (*P* < 0.001) after 2 weeks of treatment. CAM and AZM treatment reduced the bacillary load in lungs by 3.48 and 4.44 log_10_ CFU (*P* < 0.001), and in spleens by 3.51 and 5.58 log_10_ CFU (*P* < 0.001) after 4 weeks of treatment (Fig. 2). For *M. intracellulare* (ATCC 13950), the efficacy of CAM was comparable to that of AZM. CAM and AZM treatment reduced the bacillary load in lungs by 0.83 and 0.76 log_10_ CFU (*P* ≤ 0.001), and in spleen by 0.09 and 0.28 log_10_ CFU (*P* = 0.110) after 2 weeks of treatment. CAM and AZM treatment reduced the bacillary load in lungs by 1.05 and 0.99 log_10_ CFU (*P*_CAM_ < 0.001, *P*_AZM_ = 0.003), and in spleens by 0.39 and 0.56 log_10_ CFU (*P*_CAM_ = 0.003, *P*_AZM_ <0.001) after 4 weeks of treatment (Fig. 3).

### CAM treatment alleviated the degree of injury and improved the numbers of total T lymphocytes *in vivo*

**Fig 4:**
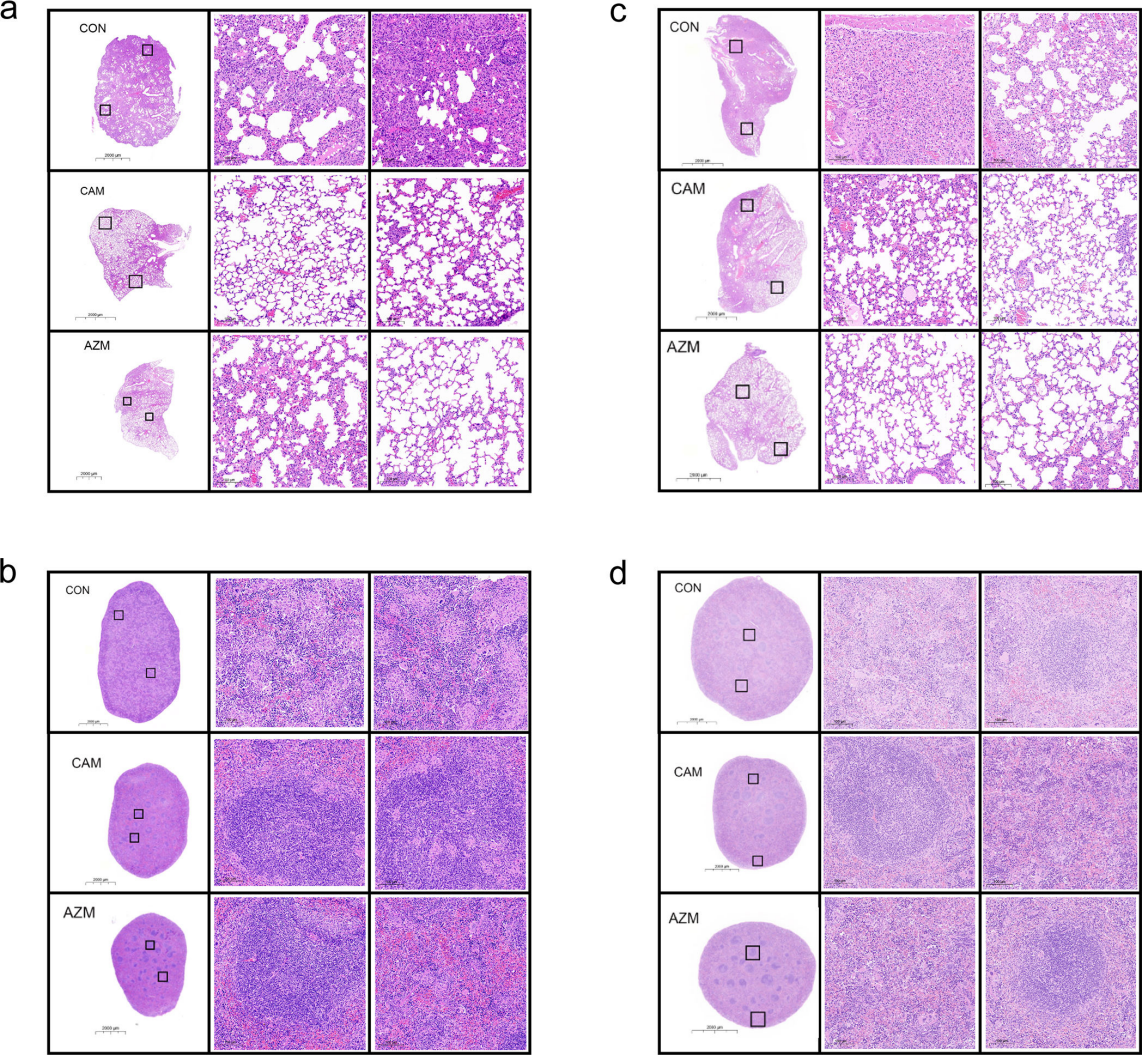
Histopathological evaluation of the lungs and spleens in BALB/c mice after 4 weeks of infection with MAC. Lungs and spleens were dissected, processed, sectioned, and stained with hematoxylin and eosin. The scale bar represents 2,000 and 100  µm. HE staining of lungs (**a**) and spleens (**b**) when BALB/c mice were infected with *M. avium* (ATCC 25291). HE staining of lungs (**c**) and spleens (**d**) when BALB/c mice were infected with *M. intracellulare* (ATCC 13950).

**Fig 5:**
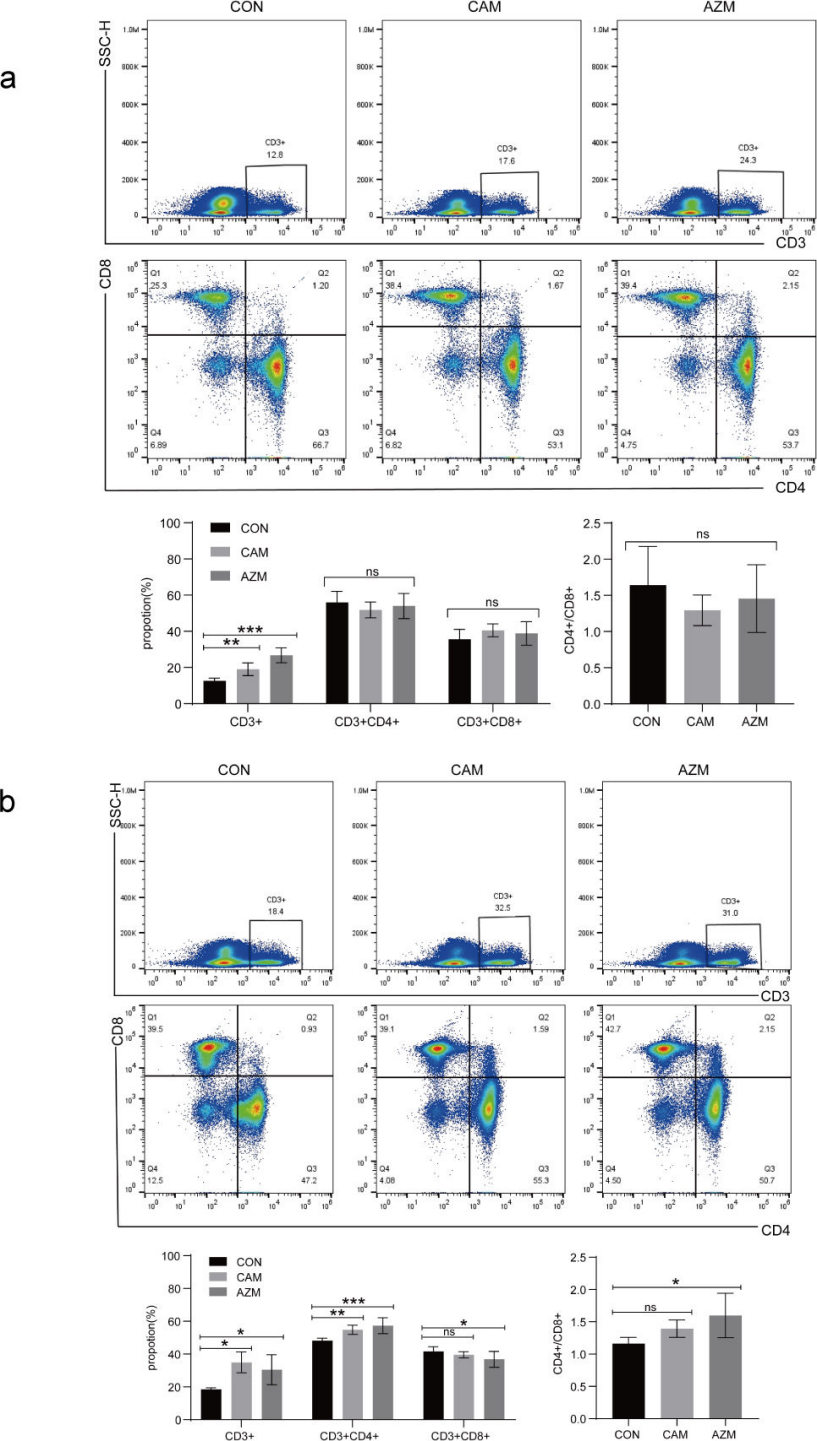
T Lymphocyte proportions in spleens of BALB/c mice after 4 weeks of infection with MAC. (**a**) BALB/c mice infected with *M. avium* (ATCC 25291) (**b**) BALB/c mice infected with *M. intracellulare* (ATCC 13950).

It was observed that CAM and AZM treatment ameliorated tissue lesions through HE staining (Fig. 4) and increased the proportion of total T lymphocytes through flow cytometry (Fig. 5). For *M. intracellulare* ATCC13950, CAM also increased the proportion of CD4^+^ T lymphocytes, without change of CD8^+^ T lymphocyte proportion and CD4^+^/CD8^+^ T lymphocyte. AZM treatment increased both CD4^+^ T lymphocyte proportion and CD4^+^/CD8^+^ T lymphocyte. For *M. avium* ATCC 25291, CAM and AZM did not influence the proportion of CD4^+^ and CD8^+^ T lymphocytes.

### CAM is synergistic with CFZ against *M. avium in vitro* and *in vivo*

CAM is compatible with nine clinically used anti-*M*. avium drugs. CAM synergized with CFZ (fractional inhibitory concentration index [FICI] = 0.5) and EMB (FICI = 0.375) to inhibit the growth of *M. avium* reference strains (ATCC 25291). Other FICI values were between 0.5 and 4, interpreted as indifference (Table 2). We further evaluated the combination of CAM and CFZ in *ex vivo* models. CAM combined with CFZ showed significant improvements over single drugs in reference strains and clinical isolates. Meanwhile, CAM combined with AMK showed similar antibacterial activity compared with single drugs (Fig. 1). This synergistic effect was also evident in the BALB/c mice model. CAM, CFZ, and CAM + CFZ treatment reduced the bacillary load by 2.15, 0.21, and 2.11 log_10_ CFU in lungs (*P* < 0.001), and by 2.82, 1.17, and 5.56 log_10_ CFU in spleens (*P* < 0.001) after 2 weeks of treatment, respectively. CAM, CFZ, and CAM + CFZ treatment reduced the bacillary load by 3.48, 1.58, and 5.66 log_10_ CFU in lungs (*P* < 0.001), and by 3.51, 2.87, and 6.24 log_10_ CFU in spleens (*P* < 0.001) after 4 weeks of treatment, respectively (Fig. 2).

**TABLE 2:** CAM is compatible with clinically used drugs against *M. avium*

Drugs	MIC alone (mg/L)	MIC in combination (mg/L)	FIC^*a*^	FICI^*b*^	Results^***c***^
CAM	8	1	1/8	0.375	Synergy
EMB	3.12	0.78	1/4		
CAM	8	2	1/4	0.5	Synergy
CFZ	0.5	0.125	1/4		
CAM	8	4	1/2	0.75	Indifference
AMK	2	0.5	1/4		
CAM	8	4	1/2	1	Indifference
RFB	0.5	0.25	1/2		
CAM	8	4	1/2	1	Indifference
BDQ	0.063	0.031	1/2		
CAM	8	4	1/2	1	Indifference
MFX	8	4	1/2		
CAM	8	4	1/2	1	Indifference
CLR	1	0.5	1/2		
CAM	8	8	1	2	Indifference
AZM	8	8	1		
CAM	8	8	1	2	Indifference
LZD	1	1	1		

^*a*^ FIC, fractional inhibitory concentration.

^*b*^ Fractional inhibitory concentration index (FICI) = MICA combination/MICA alone + MICB combination/MICB alone.

^*c*^ FICI ≤ 0.5, synergistic. 0.5 < FICI < 4, indifferent. FICI ≥ 4, antagonistic. CAM, carrimycin. EMB, ethambutol. CFZ, clofazimine. AMK, amikacin. RFB, rifabutin. LZD, linezolid. MFX, moxifloxacin. CLR, clarithromycin. AZM, azithromycin. BDQ, bedaquiline.

### The frequency of resistance in CAM was low

In *M. intracellulare*, the frequency of CAM and AZM spontaneous resistance (4× MIC) was (1.78 ± 0.16) × 10^−12^ and (3.03 ± 0.42) × 10^−12^, respectively. The results indicated that CAM is suitable for long-term administration regarding resistant frequency.

## DISCUSSION

MAC is a slowly growing mycobacterium with high pathogenicity. Infections caused by MAC are difficult to treat currently, with a success rate of only 68.1% in MAC pulmonary disease (5). Therefore, new drugs are urgently needed. CAM has been used in the treatment of acute tracheal bronchitis and acute sinusitis and shows good efficacy against common bacteria resulting in pulmonary infections. CAM has activity against some *Mycobacterium* strains in recent studies. Therefore, we explore the efficacy of CAM against MAC and firstly report the potent activity of CAM against MAC *in vivo*.

The MIC values of CAM against MAC strains are at least 8 mg/L. CAM has weaker anti-MAC activity compared with many compounds reported recently *in vitro*, such as SPR720 (6), mavintramycin A (7), and WX-081 (8). However, CAM exhibited potent activity against MAC in BALB/c mouse models, comparable to AZM. After 4 weeks of treatment, CAM and AZM reduced the lung bacillary load by 1.05 and 0.99 log_10_ CFU when mice were infected with *M. intracellulare*, and by 3.48 and 4.44 log_10_ CFU when mice were infected with *M. avium*. The *in vivo–in vitro* paradox appears to be common in macrolides. We propose two reasons to explain this phenomenon. One is the pharmacokinetic characteristics. MAC is an intracellular pathogen that survives and multiplies mainly in lungs (9). As a result, the concentrations of antimicrobial agents in lungs, particularly within alveolar macrophages (AMs), are more crucial than those in plasma. When administered intravenously at doses of 10 mg/kg for AZM, 20 mg/kg for CLR, and 5 mg/kg for erythromycin (EM) in Wistar rats, the lung to plasma concentration ratio of AZM, CLR, and EM within 24 h ranged from 44.1 to 53.4, 36.4–60.9, and 10.6–11.1, respectively (10). When orally administered at a dose of 50 mg/kg to SD rats, the AUC (from 0 to 8 h) ratio of AMs to plasma was 648 for AZM and 442 for CLR (11). The lung to plasma concentration ratio was 77 at 2.5 h and 247 at 24 h after oral administration of 80 mg/kg CAM in Wistar rats (12). This also explains that CAM and AZM exhibited activity in intracellular activity assays at concentrations lower than MIC values.

Another reason is immunomodulatory effects. Macrolides, particularly AZM, have been employed in the management of various chronic inflammatory conditions due to this property, including diffuse panbronchiolitis, cystic fibrosis, and chronic obstructive pulmonary disease (13). Immune deficiency is an important cause of MAC infections. The absolute counts of total T, CD4^+^, and CD8^+^ T lymphocytes in patients with NTM-PD were obviously lower than healthy controls (14, 15). Our results also indicated that MAC infections have a relationship with T lymphocytes. Whether CAM against MAC through improving the proportion of T lymphocytes or CAM against MAC results in the augmentation of T lymphocytes warrants further exploration. Lung damage caused by excessive inflammation is permanent and impacts lung function long after successful chemotherapy (16). In tumor patients with sepsis, CAM effectively regulates immune responses through increasing HLA-DR and CD8^+^ T cell levels (17). Meanwhile, CAM demonstrates potent anti-inflammatory efficacy and effectively mitigates organ inflammation triggered by lipopolysaccharide and cecal ligation and puncture in C57BL/6 mice through the nuclear factor kappa B (NF-κB) signaling pathway (18). In our study, treatment with CAM and AZM significantly repaired tissue damage and alleviated inflammatory cells. Tempering inflammation to mitigate subsequent tissue damage is advantageous for preserving pulmonary function and expediting symptom resolution. The promising *in vivo* activity of CAM suggests a possible role as an agent for patients infected with MAC.

Interestingly, despite close genetic and antigenic similarities, different subspecies of MAC exhibit significant variations in their interaction with host immune responses (19). *M. avium* and *M. intracellulare* demonstrate significantly distinct pathogenicity and biology in clinical features (20). In our study, although BALB/c mice infected with the same dosage by tail vein injection, a significant disparity in the pathogenic processes was observed. There is a continuous increase in the bacillary load when mice infected with *M. avium* (ATCC 25291) show a decrease followed by stabilization when mice infected with *M. intracellulare* (ATCC 13950). Treatment with CAM and AZM increased the proportion of total T cells in two models. They only enhanced the proportion of CD4^+^ T cells in mice infected with *M. intracellulare* (ATCC 13950), while showing no impact on mice infected with *M. avium* (ATCC 25291). The reasons for differences warrant further exploration.

Currently, guidelines recommend long-term multidrug therapy for upwards of 1 year to treat MAC infections. Therefore, the frequency of resistant, safety, and potential drug interactions should also be considered. In *M. intracellulare*, the frequency of CAM and AZM spontaneous resistance (4 × MIC) was (1.78 ± 0.16) × 10^−12^ and (3.03 ± 0.42) × 10^−12^, respectively, which indicated that CAM is suitable for long-term administration. As a product of genetic engineering, CAM specifically targets Spiramycin as its parent nucleus to guarantee safety. No severe adverse effects have been reported since its approval by the CFDA in 2019. Continuous oral administration of 100 mg/kg CAM for 4 weeks did not result in any adverse events in BALB/c mice. Our findings demonstrated that CAM exhibits synergistic effects with CFZ and EMB. No antagonism between CAM and drugs used in this study was observed (FICI <4). CFZ plays a pivotal role in the treatment of MAC infections, especially in those unsuitable for standard regimens (21, 22). Many studies suggest replacing RIF with CFZ in the treatment regimen (23–25) since RIF can reduce peak plasma concentrations of CLR/AZM by inducing cytochrome P450 isoenzymes (26). Previous research has reported CFZ combined with CLR shows a great improvement in efficacy in mouse models of *M. avium* and *M. tuberculosis* (27, 28). EMB is a common drug used in the treatment of MAC infections. Previous studies show that EMB synergizes with macrolide (29). Our study also found that EMB synergized with CAM against *M. avium* ATCC 25291. However, the interaction between CAM and EMB against *M. intracellulare* ATCC 13950 was interpreted as indifference. Considering the efficacy and drug-drug interactions of CAM, we will further explore regimens containing CAM to provide evidence for its clinical application.

Based on current research on CAM, the multifaceted biological activities, such as anti-inflammatory (18), anti-viral (30), and anti-tumor properties (31, 32), are unique advantages of CAM. CAM reduces the efficiency of programmed −1 ribosomal frameshifting in coronaviruses, thereby impeding viral replication in a broad-spectrum manner (33). CAM accelerates the degradation of selenoprotein H, leading to oxidative stress, disrupted ribosomal biogenesis, and apoptosis in tumor cells (34). The pharmacological activities of CAM enable it to be better applied in complex clinical scenarios (3, 35).

Our study also has some limitations. First, we only evaluate the activities against reference strains *in vivo*. In our future studies, more species of MAC clinical isolates, especially drug-resistant strains, will be used. Second, we only focused on T cells; we will further explore the impact on other immune cells. Third, we did not conduct experiments related to pharmacokinetics and mechanism of resistance due to the absence of a single component.

In conclusion, CAM was highly effective in inhibiting the growth of MAC *in vitro* and *in vivo*. Its low frequency of resistant, safety, immunoregulatory properties combined with the synergistic effect with CFZ are beneficial for the treatment of MAC infections. As such, CAM represents a potential candidate into novel therapeutic anti-MAC regimens.

## MATERIALS AND METHODS

### Strains

*M. avium* reference strain (ATCC 25291), *M. intracellulare* reference strain (ATCC 13950), and 15 clinical isolates were obtained from the National Clinical Laboratory on Tuberculosis, Beijing Chest Hospital. Strains were frozen in 1 mL aliquots and stored at −80°C before use. For each experiment, an aliquot was thawed and subcultured in Middlebrook 7H9 broth (Difco) supplemented with 10% (vol/vol) oleic acid-albumin-dextrose-catalase (OADC) (Becton Dickinson), 0.2% (vol/vol) glycerol, and 0.05% Tween 80 at 37℃.

### Compounds

Carrimycin was sourced from Shenyang Tonglian Group Co., Ltd. (Shanghai, China). Azithromycin was purchased from TargetMol. Dexamethasone was purchased from Biochempartner Co., Ltd.

### Minimum inhibitory concentration (MIC) measurements

The MICs of CAM and AZM against *M. avium*, *M. intracellulare* reference strains, and clinical strains were tested according to Clinical and Laboratory Standards Institute (CLSI) guidelines. We not only used cation-adjusted Mueller-Hinton broth (CAMHB) as guidelines recommended but also used Middlebrook 7H9 broth as previously described (36).

### FICI determination

Synergy between CAM and ethambutol, clofazimine, amikacin, rifabutin, linezolid, moxifloxacin, clarithromycin, azithromycin, and bedaquiline was assessed against *M. avium* reference strain (ATCC 25291) using broth microdilution checkerboard titration technique. All synergy tests were performed in CAMHB. Synergy test results were interpreted based upon the fractional inhibitory concentration index (FICI), FICI = MIC_A combination_/MIC_A alone_ + MIC_B combination_/MIC_B alone_. Drug interactions were classified as synergistic (FICI ≤0.5), indifferent (0.5 < FICI ≤ 4), and antagonistic (FICI >4.0).

### The frequency of resistance

Log-phase M*. intracellulare* ATCC 13950 was washed three times and resuspended in PBS. Then, 100 µL of resuspended *M. intracellulare* ATCC 13950 (1 × 10^9^ CFU) was spread on 7H10 agar with and without 4× MIC CAM or AZM and cultured for 4 weeks at 37℃. This experiment was repeated four times. The result was calculated as follows: the frequency of resistance = the bacillary load in 7H10 agar containing 4× MIC CAM or AZM/the bacillary load in 7H10 agar.

### MTT assay

Drugs were threefold diluted in 96-well microtiter plates and co-incubated with J774A.1 cells at 4 × 10^5^ per mL in DMEM medium containing 10% fetal bovine serum (FBS) for 48 h. Then, cells were incubated for 4 h with 0.8 mg/mL of MTT (3-[4,5-dimethylthiazol-2-yl]−2,5-diphenyltetrazolium bromide). Washing with phosphate-buffered saline (PBS) was followed by the addition of DMSO, gentle shaking for 5 min so that complete dissolution was achieved. Absorbance was recorded at 560 nm using the microplate spectrophotometer system.

### Intracellular activity assay

J774A.1 cells were infected with MAC (multiplicity of infection, MOI = 1 when tested the effect of single drugs and MOI = 3 when tested the effect of combination). After 6 h of incubation at 37°C, 5% CO_2_, cells were washed three times with PBS to remove extracellular mycobacteria. Then, fresh DMEM containing 10% FBS and 2 mg/L drugs or the same volume of DMSO was added and incubated for 48 h. After that, J774A.1 cells were lysed with 200 µL of 0.1% sodium dodecyl sulfate (SDS), diluted with fresh media, and plated onto 7H10 plates supplemented with 10% OADC to measure the CFU.

### Efficacy of CAM in MAC-infected BALB/c mice

Animal experiments were performed with 6–8 weeks female BALB/c mice, purchased from Beijing Vital River Laboratory Animal Technology Co., Ltd. (Beijing, China). A total of 126 BALB/c mice were continuously administered dexamethasone (5 mg/kg) by gavage for 7 days before infection. Then, 75 mice were infected with *M. avium* reference strain (ATCC 25291), and 51 mice were infected with *M. intracellulare* reference strain (ATCC 13950) at 2 × 10^6^ CFU/mice by tail vein injection. Six mice were euthanized 1 day after infection, and the remaining mice were divided into different groups randomly. The doses of CAM, AZM, and CFZ administered were 100, 100, and 25 mg/kg, respectively, via gavage. Then, 10-fold serial dilutions of lung and spleen homogenates were inoculated onto 7H10-OADC agar. The bacterial content was estimated from the number of colonies that grew on plates incubated for 21 days at 37℃, 5% CO_2_ incubator. Three lungs and spleens of mice from each group were randomly selected, dissected, and fixed with 4% paraformaldehyde (PFA) for H&E staining.

### Flow cytometry

Six spleens of each group were randomly selected for flow cytometry. The method for isolating single cells was according to the manufacturer’s instrument (thermofisher.cn/immunology). Briefly, dissect and grind the spleen, cell suspensions were passed through a 70 µm Falcon nylon cell strainer (Corning, NY, USA). Then, cells were incubated with 2 mL RBC lysis buffer for 5 min on ice. Cells were re-suspended in PBS and stained with Fixable Viability Dye eFluor 780, anti-mouse CD3, anti-mouse CD4, and anti-mouse CD8 in accordance with the manufacturer’s instructions.

### Data analysis

Statistical Product and Service Solutions (SPSS) 23.0 was employed for statistical analysis, GraphPad Prism 8.0 was utilized for plotting, and measurement data were presented as mean ± standard deviation ($\overset{-}{x}\pm s$). CFU counting was analyzed subsequent to logarithmic transformation. Normality testing was conducted initially for the data. Independent sample *t*-test or one-way ANOVA was adopted for data with normal distribution; non-parametric tests were applied for non-normal distribution data with 2 or k samples. Statistical significance was regarded when *P* < 0.05.
